# *N*^6^-Methyladenosine and Reader Protein YTHDF2 Enhance the Innate Immune Response by Mediating DUSP1 mRNA Degradation and Activating Mitogen-Activated Protein Kinases during Bacterial and Viral Infections

**DOI:** 10.1128/mbio.03349-22

**Published:** 2023-01-10

**Authors:** Jian Feng, Wen Meng, Luping Chen, Xinquan Zhang, Ashley Markazi, Weiming Yuan, Yufei Huang, Shou-Jiang Gao

**Affiliations:** a Cancer Virology Program, UPMC Hillman Cancer Center, University of Pittsburgh, Pittsburgh, Pennsylvania, USA; b Department of Microbiology and Molecular Genetics, University of Pittsburgh, Pittsburgh, Pennsylvania, USA; c Department of Molecular Microbiology and Immunology, Keck School of Medicine, University of Southern California, Los Angeles, California, USA; d Department of Electrical and Computer Engineering, University of Pittsburgh, Pittsburgh, Pennsylvania, USA; e Department of Biomedical Informatics, University of Pittsburgh, Pittsburgh, Pennsylvania, USA; f Department of Medicine, University of Pittsburgh, Pittsburgh, Pennsylvania, USA; Virginia Polytechnic Institute and State University

**Keywords:** *N*^6^-methyladenosine, m^6^A, YTHDF2, innate immunity, dual-specificity phosphatase 1, DUSP1, mitogen-activated protein kinases, MAPKs, p38, JNK, p38 kinases

## Abstract

Mitogen-activated protein kinases (MAPKs) play critical roles in the induction of numerous cytokines, chemokines, and inflammatory mediators that mobilize the immune system to counter pathogenic infections. Dual-specificity phosphatase 1 (DUSP1) is a member of the dual-specificity phosphatases that inactivates MAPKs through a negative-feedback mechanism. Here, we report that in response to viral and bacterial infections, not only the DUSP1 transcript but also its *N*^6^-methyladenosine (m^6^A) levels rapidly increase together with that of the m^6^A reader protein YTHDF2, resulting in enhanced YTHDF2-mediated DUSP1 transcript degradation. The knockdown of DUSP1 promotes p38 and Jun N-terminal kinase (JNK) phosphorylation and activation, thus increasing the expression of innate immune response genes, including the interleukin-1β (IL-1β), colony-stimulating factor 3 (CSF3), transglutaminase 2 (TGM2), and proto-oncogene tyrosine-protein kinase Src (SRC) genes. Similarly, the knockdown of the m^6^A eraser ALKBH5 increases the DUSP1 transcript m^6^A level, resulting in accelerated transcript degradation, the activation of p38 and JNK, and the enhanced expression of IL-1β, CSF3, TGM2, and SRC. These results demonstrate that m^6^A and the reader protein YTHDF2 orchestrate optimal innate immune responses during viral and bacterial infections by downregulating the expression of a negative regulator, DUSP1, of the p38 and JNK pathways that are central to innate immune responses against pathogenic infections.

## INTRODUCTION

The innate immune system is a highly efficient cellular and molecular network in mammalian cells that protects the organism against pathogenic infections (1). This first line of defense against invasion is achieved by sensing the pathogens through pattern recognition receptors (2). The stimulation of pattern recognition receptors on the cell surface and in the cytoplasm of innate immune cells activates multiple mitogen-activated protein kinases (MAPKs), including the extracellular signal-regulated kinase (ERK), p38, and the Jun N-terminal kinase (JNK) (3). MAPKs are a group of highly conserved serine/threonine protein kinases in eukaryotes (4), which play critical roles in inducing numerous cytokines, chemokines, and inflammatory mediators that mobilize the immune system to counter pathogenic infections (5). Furthermore, the induction of a proinflammatory response promotes the recruitment of additional immune cells to invoke secondary innate and adaptive immune responses (6).

Dual-specificity phosphatase 1 (DUSP1) (also known as MAPK phosphatase 1 [MKP-1]) was initially identified in cultured murine cells (7). It is a member of the DUSPs, which are key players in inactivating different MAPKs (8). DUSP1 expression is enhanced upon numerous pathogenic infections, and it is an important feedback mechanism for controlling excessive immune responses and inflammation (9, 10). By dephosphorylation, DUSP1 inhibits the activation of specific threonine and tyrosine residues on p38 and JNK, resulting in the inactivation of the inflammatory or innate immune response by inhibiting the expression of numerous effector genes at the transcriptional or posttranscriptional level (11).

*N*^6^-methyladenosine (m^6^A), a dynamic posttranscriptional RNA modification, is critical for almost all aspects of RNA metabolism and functions, including structure, maturation, stability, splicing, export, translation, and decay (12). Recent studies show that m^6^A modification not only directly regulates the expression of innate immune response genes but also indirectly affects the mRNA metabolism pathway to further regulate the innate immune response during bacterial and viral infections (13–16).

We have previously shown that m^6^A plays an important role in regulating the innate immune response against both bacterial and viral infections by directly and indirectly regulating the expression of innate immune response genes (13). More recent work indicates that m^6^A is a vital factor for regulating the innate immune response and cytokines by affecting the IκB kinase ε (IKKε)/TANK-binding kinase 1 (TBK1)/interferon regulatory factor 3 (IRF3), MAPK, and NF-κB pathways (17, 18). In this study, we have discovered that DUSP1 is a direct m^6^A target, and m^6^A and the reader protein YTHDF2 regulate DUSP1 stability to maximize the innate immune response during bacterial and viral infections.

## RESULTS

### m^6^A mediates DUSP1 transcript expression during bacterial infection.

**FIG 1:**
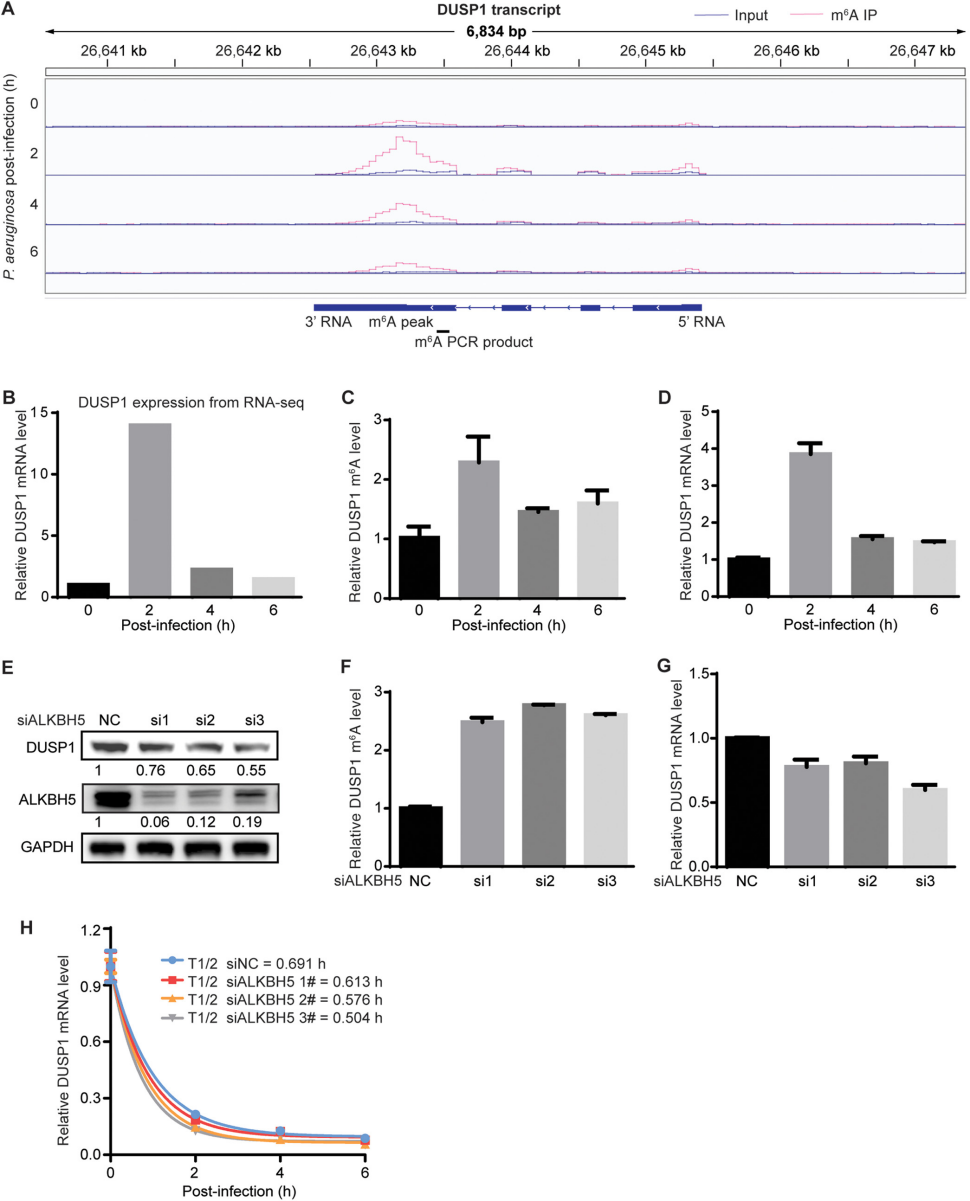
m^6^A mediates DUSP1 transcript stability during bacterial infection in RAW264.7 cells. (A) Tracks of m^6^A peaks on the DUSP1 transcript 0, 2, 4, and 6 h after infection with P. aeruginosa. (B) Expression levels of the DUSP1 transcript 0, 2, 4, and 6 h after infection with P. aeruginosa quantified by transcriptome sequencing (RNA-seq). (C) m^6^A levels on the DUSP1 transcript 0, 2, 4, and 6 h after infection with P. aeruginosa examined by MeRIP-qPCR. (D) Expression levels of the DUSP1 transcript 0, 2, 4, and 6 h after infection with P. aeruginosa quantified by RT-qPCR. (E) Examination of DUSP1 and ALKBH5 protein levels following ALKBH5 knockdown in RAW264.7 cells by Western blotting. (F) m^6^A levels on the DUSP1 transcript following ALKBH5 knockdown in RAW264.7 cells examined by MeRIP-qPCR. (G) Expression levels of the DUSP1 transcript following ALKBH5 knockdown examined by RT-qPCR. (H) Alterations in the half-lives (T1/2) of the DUSP1 transcript following ALKBH5 knockdown during P. aeruginosa infection examined by RT-qPCR at the indicated time points following the addition of 10 μg/mL actinomycin D. siALKBH5, siRNA targeting ALKBH5.

We have previously mapped the cellular expression profiles and m^6^A epitranscriptomes and identified a set of genes, including innate immune response genes, that are differentially methylated and differentially expressed during viral and bacterial infections (13). Among them, DUSP1, an important regulator of innate immune response genes, was significantly hypermethylated during infection with the Gram-negative bacterium Pseudomonas aeruginosa, which peaked at 2 h postinfection (hpi) and then decreased at 4 and 6 hpi (Fig. 1A). At the same time, DUSP1 transcript expression was upregulated, which also peaked at 2 hpi and then decreased at 4 and 6 hpi (Fig. 1B). These results were consistent with the induction of DUSP1 by lipopolysaccharide (LPS) or Toll-like receptor (TLR) ligands reported in previous studies (19, 20).

We confirmed the increase of DUSP1 transcript m^6^A during P. aeruginosa infection by m^6^A immunoprecipitation (m^6^A-IP) reverse transcription–quantitative real-time PCR (MeRIP-qPCR). The DUSP1 transcript m^6^A level was increased by 2.3-fold 2 h after infection with P. aeruginosa but then decreased at 4 and 6 hpi (Fig. 1C). Reverse transcription–quantitative real-time PCR (RT-qPCR) further confirmed the increased DUSP1 transcript expression following P. aeruginosa infection, which peaked at 2 hpi (Fig. 1D).

We then performed knockdown of ALKBH5, an m^6^A “eraser,” to determine whether the increase of DUSP1 transcript m^6^A could affect its expression (Fig. 1E). As expected, ALKBH5 knockdown further increased the m^6^A level of the DUSP1 transcript by 2.5- to 2.8-fold (Fig. 1F). However, DUSP1 transcript expression was reduced by 25% to 40% (Fig. 1G), which was also reflected in the decreased DUSP1 protein level (Fig. 1E). These results suggest that the increased m^6^A level during P. aeruginosa infection likely serves to reverse the upregulation of the DUSP1 transcript. Since one of the functions of m^6^A modification is to mediate RNA decay (21, 22), we examined DUSP1 transcript stability during P. aeruginosa infection. The half-life of the DUSP1 transcript was reduced by 12.7% to 37.1% following ALKBH5 knockdown (Fig. 1H), indicating m^6^A regulation of DUSP1 RNA decay during P. aeruginosa infection.

### YTHDF2 mediates m^6^A-dependent DUSP1 transcript degradation.

In order to further delineate the role of m^6^A in the innate immune response, we infected mouse RAW264.7 macrophage cells with different doses of Gram-negative or -positive bacteria or human herpes simplex virus 1 (HSV-1) and examined the expression of innate immune response genes (see Fig. S1 in the supplemental material). Infection with 10^7^ bacteria of the Gram-positive organism Corynebacterium diphtheriae, 10^7^ bacteria of the Gram-negative organism P. aeruginosa, or HSV-1 at a multiplicity of infection (MOI) of 1 induced the maximum expression of innate immune response genes, including colony-stimulating factor 3 (CSF3), interleukin-1β (IL-1β), transglutaminase 2 (TGM2), and proto-oncogene tyrosine-protein kinase Src (SRC), under our experimental conditions (Fig. S1). Thus, we used these conditions in subsequent experiments.

10.1128/mbio.03349-22.1 FIG S1 Expression of the CSF3, IL-1β, TGM2, and SRC transcripts following infection with different doses of C. diphtheriae, P. aeruginosa, or HSV-1 in RAW264.7 cells at the indicated time points examined by RT-qPCR. Download FIG S1, TIF file, 1.3 MB. Copyright © 2023 Feng et al. 2023 Feng et al. https://creativecommons.org/licenses/by/4.0/ This content is distributed under the terms of the Creative Commons Attribution 4.0 International license.

**FIG 2:**
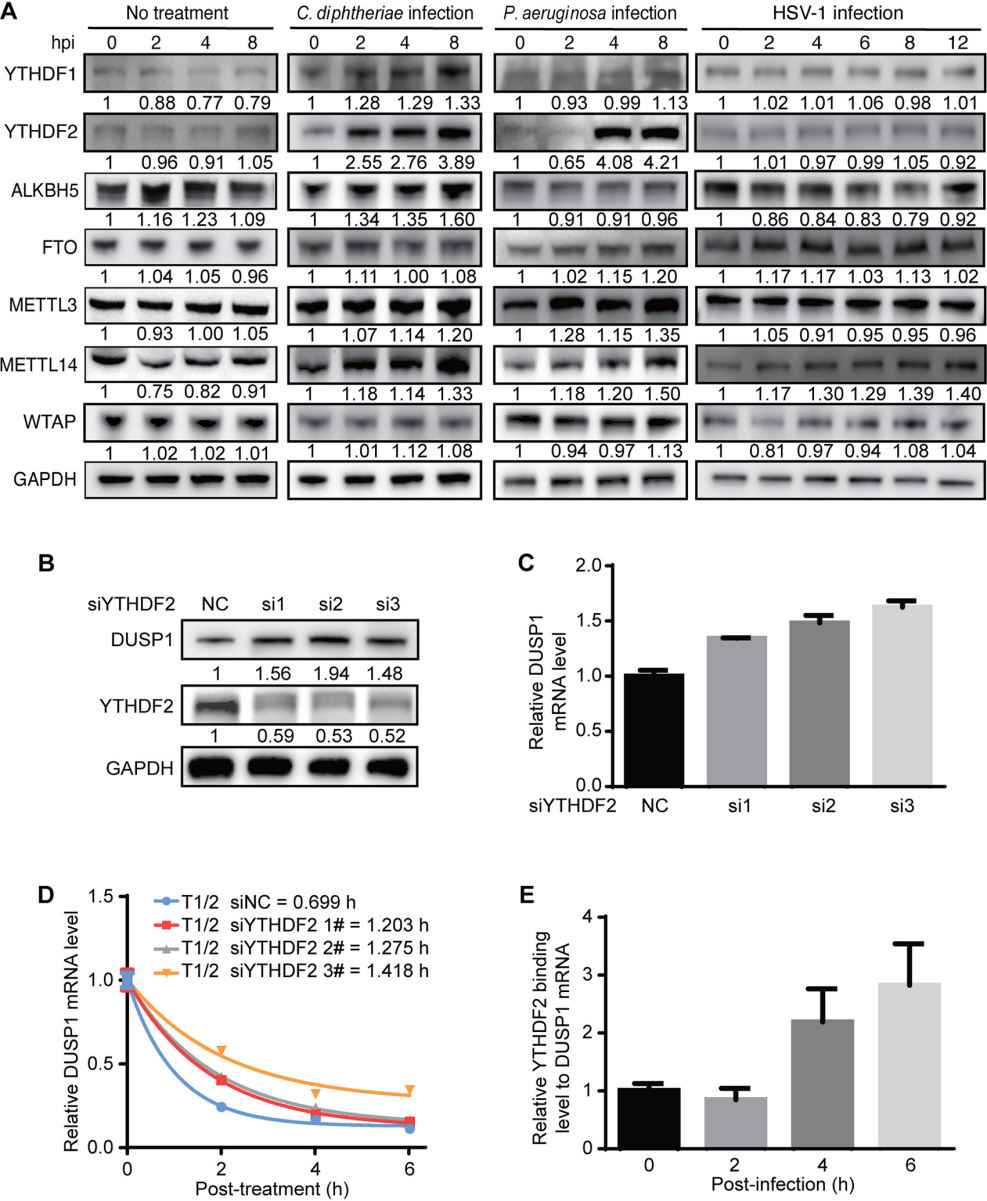
YTHDF2 mediates m^6^A-dependent DUSP1 transcript stability during bacterial and viral infections in RAW264.7 cells. (A) Protein levels of the m^6^A writers METTL3, METTL14, and WTAP; the erasers ALKBH5 and FTO; and the readers YTHDF1 and YTHDF2 with or without infection by C. diphtheriae, P. aeruginosa, or HSV-1 at the indicated time points examined by Western blotting. (B) Examination of DUSP1 and YTHDF2 protein levels following YTHDF2 knockdown by Western blotting. (C) Expression levels of the DUSP1 transcript following YTHDF2 knockdown examined by RT-qPCR. (D) Alterations of the half-lives of the DUSP1 transcript following YTHDF2 knockdown in RAW264.7 cells during P. aeruginosa infection examined by RT-qPCR at the indicated time points following the addition of 10 μg/mL actinomycin D. (E) Binding of YTHDF2 to the DUSP1 transcript at the indicated time points following P. aeruginosa infection examined by RIP-qPCR.

We examined the protein levels of m^6^A “writers,” “erasers,” and “readers” during bacterial and viral infections (Fig. 2A). The m^6^A writer protein METTL14 showed marginal increases during P. aeruginosa, C. diphtheriae, and wild-type (WT) HSV-1 infections, while another m^6^A writer protein, METTL3, showed marginal increases during P. aeruginosa and C. diphtheriae infections (Fig. 2A). The eraser protein ALKBH5 also showed a slight increase during C. diphtheriae infection. Of the reader proteins examined, YTHDF1 showed a marginal increase during C. diphtheriae infection. However, YTHDF2 showed significant increases during C. diphtheriae and P. aeruginosa infections, by 3.89- and 4.21-fold, respectively, at 8 hpi, respectively (Fig. 2A). Since YTHDF2 mediates m^6^A-dependent RNA decay (23), we performed knockdown of YTHDF2 (Fig. 2B) and observed an upregulation of the DUSP1 transcript (Fig. 2C), which was also reflected in an increase in the DUSP1 protein level (Fig. 2B). The knockdown of YTHDF2 almost doubled the half-life of the DUSP1 transcript (Fig. 2D). Furthermore, YTHDF2 RNA immunoprecipitation reverse transcription–quantitative real-time PCR (RIP-qPCR) showed the binding of YTHDF2 protein to the DUSP1 RNA transcript, which was significantly increased at 4 and 6 hpi (Fig. 2E), correlating with the increased YTHDF2 protein level at these time points (Fig. 2A). Together, these results indicate that the upregulation of the YTHDF2 protein promotes the degradation of the DUSP1 transcript during P. aeruginosa infection.

### DUSP1 regulates p38 and JNK phosphorylation during bacterial and viral infections.

**FIG 3:**
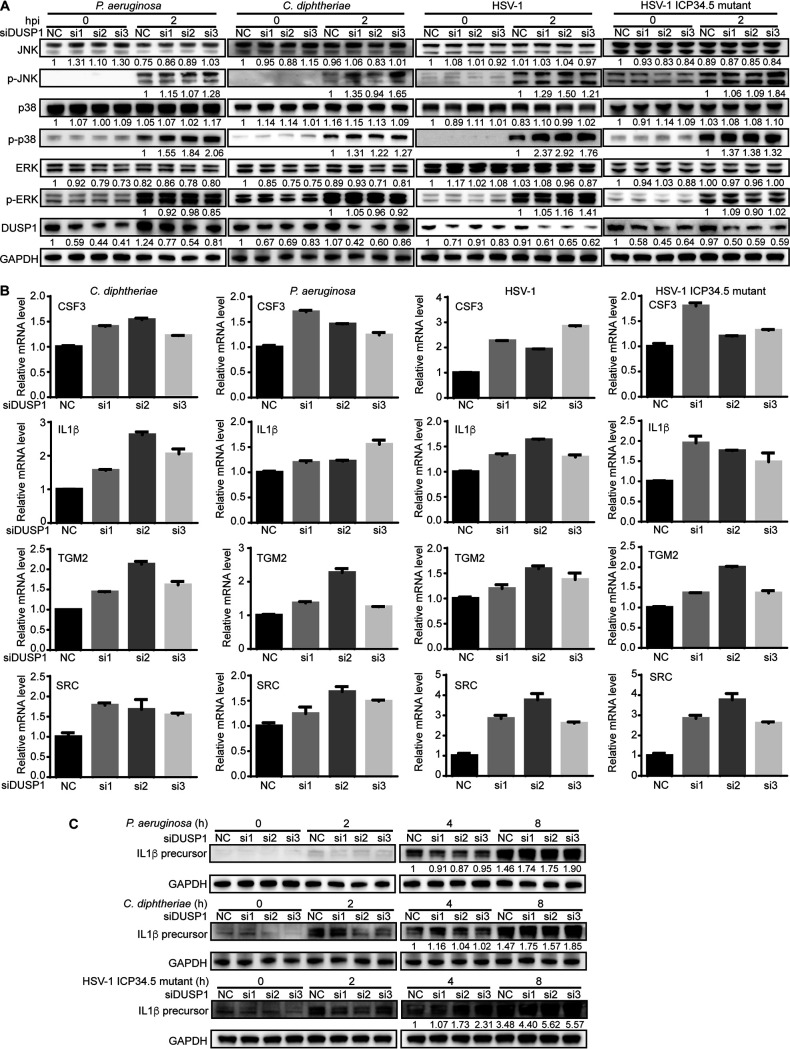
DUSP1 regulates p38 and JNK phosphorylation and the expression of innate immune response genes during bacterial and viral infections in RAW264.7 cells. (A) DUSP1 knockdown enhanced p38 and JNK phosphorylation during infection by P. aeruginosa, C. diphtheriae, HSV-1, or the HSV-1 ICP34.5 mutant. (B) DUSP1 knockdown enhanced the expression of the IL-1β, CSF3, TGM2, and SRC genes during infection by P. aeruginosa, C. diphtheriae, HSV-1, or the HSV-1 ICP34.5 mutant. (C) DUSP1 knockdown enhanced the protein level of IL-1β during infection by P. aeruginosa, C. diphtheriae, or the HSV-1 ICP34.5 mutant.

As an important innate immune response gene, DUSP1 inactivates MAPKs by inhibiting their phosphorylation (11). Our results showed the upregulation of the DUSP1 transcript during bacterial and viral infections, which was reversed by m^6^A- and YTHDF2-mediated transcript degradation (Fig. 1 and 2). As expected, the ERK, p38, and JNK MAPKs were activated at 2 hpi by P. aeruginosa, C. diphtheriae, WT HSV-1, and the HSV-1 ICP34.5 mutant (Fig. 3A). We included the HSV-1 ICP34.5 mutant because the ICP34.5 protein has been shown to prevent the induction of innate immune response genes during HSV-1 infection by directly inhibiting TBK1 activation and eukaryotic initiation factor 2α (eIF2α) function (24, 25). To determine whether DUSP1 regulated the activation of MAPKs during bacterial and viral infections, we performed DUSP1 knockdown. Western blot results showed that the levels of phosphorylated p38 (p-p38) and p-JNK were increased following DUSP1 knockdown during bacterial and viral infections (Fig. 3A). The activation of MAPKs can induce their downstream transcriptional factors, including AP-1 and C/EBP, resulting in the upregulation of target genes, including numerous innate immune response genes (26). Consistent with the increased levels of p-p38 and p-JNK following DUSP1 knockdown, the levels of CSF3, IL-1β, TGM2, and SRC transcripts were upregulated (Fig. 3B). We observed some variations in the effects of different DUSP1 small interfering RNAs (siRNAs) on the expression of the IL-1β, CSF3, TGM2, and SRC transcripts. These might be due to the different knockdown kinetics of these siRNAs. The IL-1β protein level was also upregulated after DUSP1 knockdown during infections by P. aeruginosa, C. diphtheriae, and the HSV-1 ICP34.5 mutant (Fig. 3C). However, the upregulation of the IL-1β protein was weak during WT HSV-1 infection, and its increase was only marginal after DUSP1 knockdown (Fig. S2A), which was likely due to the inhibition of the innate immune response by the HSV-1 ICP34.5 protein (24, 25). These results indicated that DUSP1 inhibited p-p38 and p-JNK activation to block the innate immune response during bacterial and viral infections.

10.1128/mbio.03349-22.2 FIG S2 Protein level of the IL-1β precursor following knockdown of DUSP1 (A) or ALKBH5 (B) at different time points following HSV-1 infection in RAW264.7 cells. Download FIG S2, TIF file, 1.1 MB. Copyright © 2023 Feng et al. 2023 Feng et al. https://creativecommons.org/licenses/by/4.0/ This content is distributed under the terms of the Creative Commons Attribution 4.0 International license.

### ALKBH5 regulates p38 and JNK phosphorylation and their downstream innate immune response genes during bacterial and viral infections.

**FIG 4:**
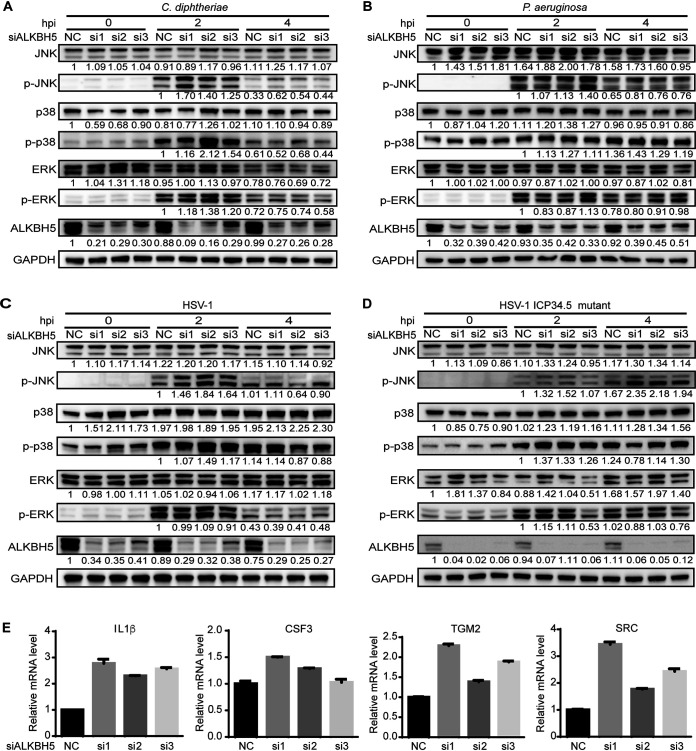
ALKBH5 regulates p38 and JNK phosphorylation and the transcription of innate immune response genes during bacterial and viral infections in RAW264.7 cells. (A to D) ALKBH5 knockdown enhanced p38 and JNK phosphorylation during infection with C. diphtheriae (A), P. aeruginosa (B), HSV-1 (C), or the HSV-1 ICP34.5 mutant (D). (E) *De novo* transcription of the IL-1β, CSF3, TGM2, and SRC genes following ALKBH5 knockdown 2 h after infection with P. aeruginosa examined by a nuclear run-on assay. Cells treated with 4-thiouridine for 1 h after ALKBH5 knockdown were infected with P. aeruginosa for 4 h and collected for a nuclear run-on assay.

Since DUSP1 inactivated p38 and JNK during bacterial and viral infections, and ALKBH5 knockdown reduced DUSP1 transcript stability by increasing the m^6^A level, we examined ALKBH5’s regulation of p38 and JNK activation. ALKBH5 knockdown increased the levels of p-p38 and p-JNK during infection by P. aeruginosa, C. diphtheriae, WT HSV-1, or the HSV-1 ICP34.5 mutant (Fig. 4A to D). Some minor increases in p-ERK were also observed 2 h after infection with C. diphtheriae. Since the increased p-p38 and p-JNK levels could lead to an enhanced activation of their downstream transcriptional factors, we examined the *de novo* transcription of the target genes by performing a nuclear run-on assay during P. aeruginosa infection. ALKBH5 knockdown indeed increased the transcriptional activities of the IL-1β, CSF3, TGM2, and SRC genes (Fig. 4E).

**FIG 5:**
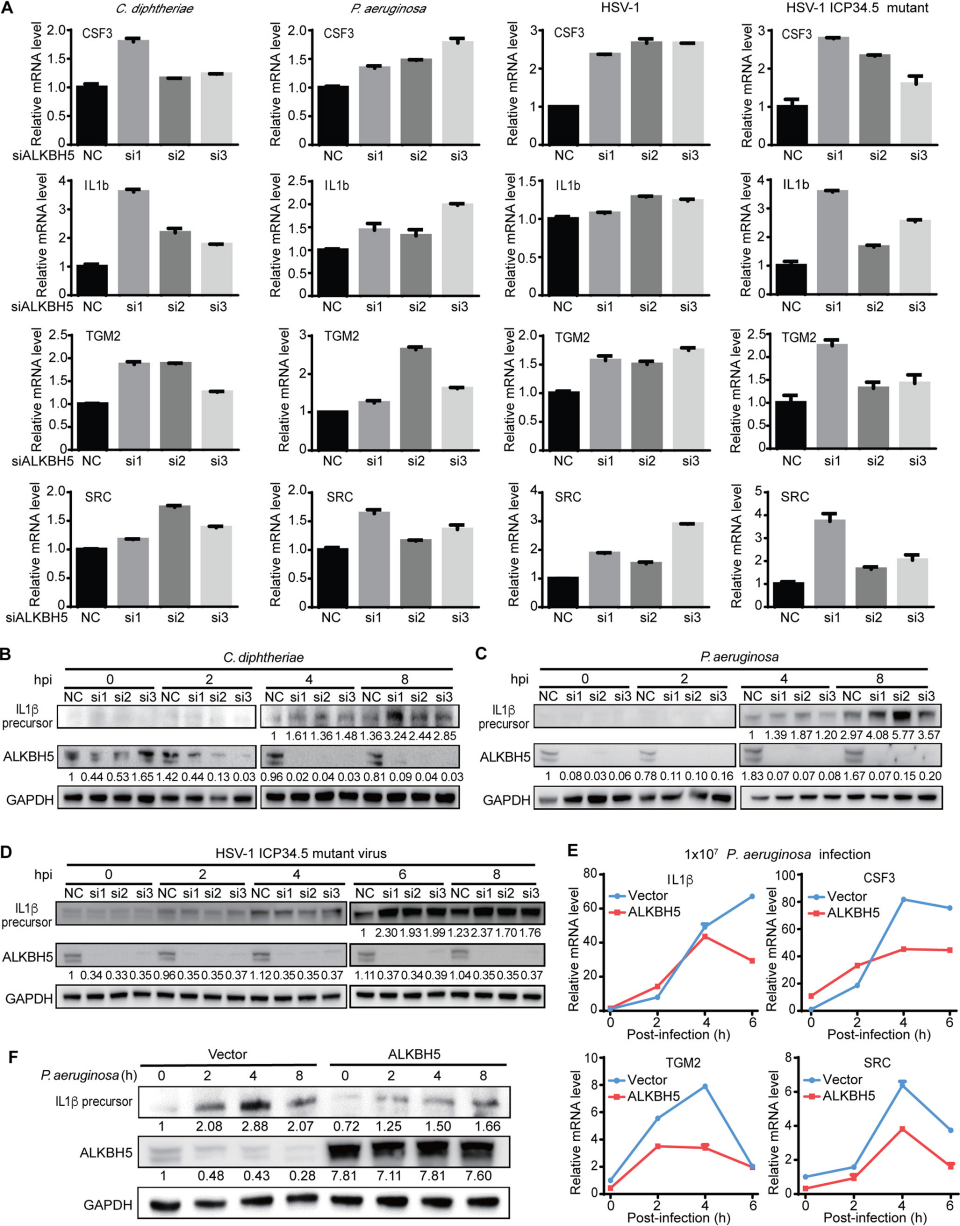
ALKBH5 regulates the expression of innate immune response genes during bacterial and viral infections in RAW264.7 cells. (A) ALKBH5 knockdown enhanced the expression levels of the IL-1β, CSF3, TGM2, and SRC transcripts during P. aeruginosa, C. diphtheriae, HSV-1, or HSV-1 ICP34.5 mutant infection. (B to D) ALKBH5 knockdown enhanced the protein level of IL-1β during infection by C. diphtheriae (B), P. aeruginosa (C), or the HSV-1 ICP34.5 mutant (D). (E) ALKBH5 overexpression inhibited the expression of the IL-1β, CSF3, TGM2, and SRC genes during P. aeruginosa infection as measured by RT-qPCR. (F) ALKBH5 overexpression inhibited the protein level of IL-1β during P. aeruginosa infection as measured by Western blotting.

We further examined the role of ALKBH5 in the expression of innate immune response genes. ALKBH5 knockdown increased the levels of the IL-1β, CSF3, TGM2, and SRC transcripts during infection by P. aeruginosa, C. diphtheriae, WT HSV-1, or the ICP34.5 mutant virus (Fig. 5A). Similar to DUSP1 knockdown, we noticed variations of the effects of different ALKBH5 siRNAs on both the transcription and expression of the IL-1β, CSF3, TGM2, and SRC genes (Fig. 4E and Fig. 5). These variations might be due to the different knockdown kinetics of these siRNAs, which might impact the m^6^A level of the DUSP1 transcript, the DUSP1 expression level, and p-p38 and p-JNK levels, leading to variable transcription and expression levels of these downstream genes.

The protein level of IL-1β was also upregulated after ALKBH5 knockdown during infections by P. aeruginosa, C. diphtheriae, and the HSV-1 ICP34.5 mutant (Fig. 5B to D). However, the upregulation of the IL-1β protein was marginal during WT HSV-1 infection (Fig. S2B). In contrast, the overexpression of ALKBH5 reduced the levels of the IL-1β, CSF3, TGM2, and SRC transcripts (Fig. 5E) and downregulated the IL-1β protein level (Fig. 5F) during P. aeruginosa infection. It was interesting that the reduced expression of the four transcripts had different kinetics following the overexpression of ALKBH5 (Fig. 5E). The effect of ALKBH5 overexpression was observed for TGM2 and SRC transcripts by as early as 2 hpi, which disappeared by 6 hpi. However, the effect was not observed for CSF3 until 4 hpi and for IL-1β until 6 hpi. It is possible that the promoters of these genes might endow them with different kinetics in response to the activation of the p38 and JNK pathways.

Because our results showed an important role of ALKBH5 in regulating the innate immune response, we further examined the impact of ALKBH5 knockdown on HSV-1 replication. ALKBH5 knockdown reduced the replication of WT HSV-1 or the ICP34.5 mutant virus (Fig. S3). These results are in agreement with those of a previous study showing reduced HSV-1 replication after ALKBH5 knockout (14).

10.1128/mbio.03349-22.3 FIG S3 ALKBH5 knockdown inhibits HSV-1 replication. RAW264.7 cells transfected with ALKBH5 short hairpin RNAs (shRNAs) or a scrambled control (NC) were infected with HSV-1 at an MOI of 1 for 48 h, and the supernatants were collected for plaque assays to determine the viral titers. Download FIG S3, TIF file, 0.3 MB. Copyright © 2023 Feng et al. 2023 Feng et al. https://creativecommons.org/licenses/by/4.0/ This content is distributed under the terms of the Creative Commons Attribution 4.0 International license.

**FIG 6:**
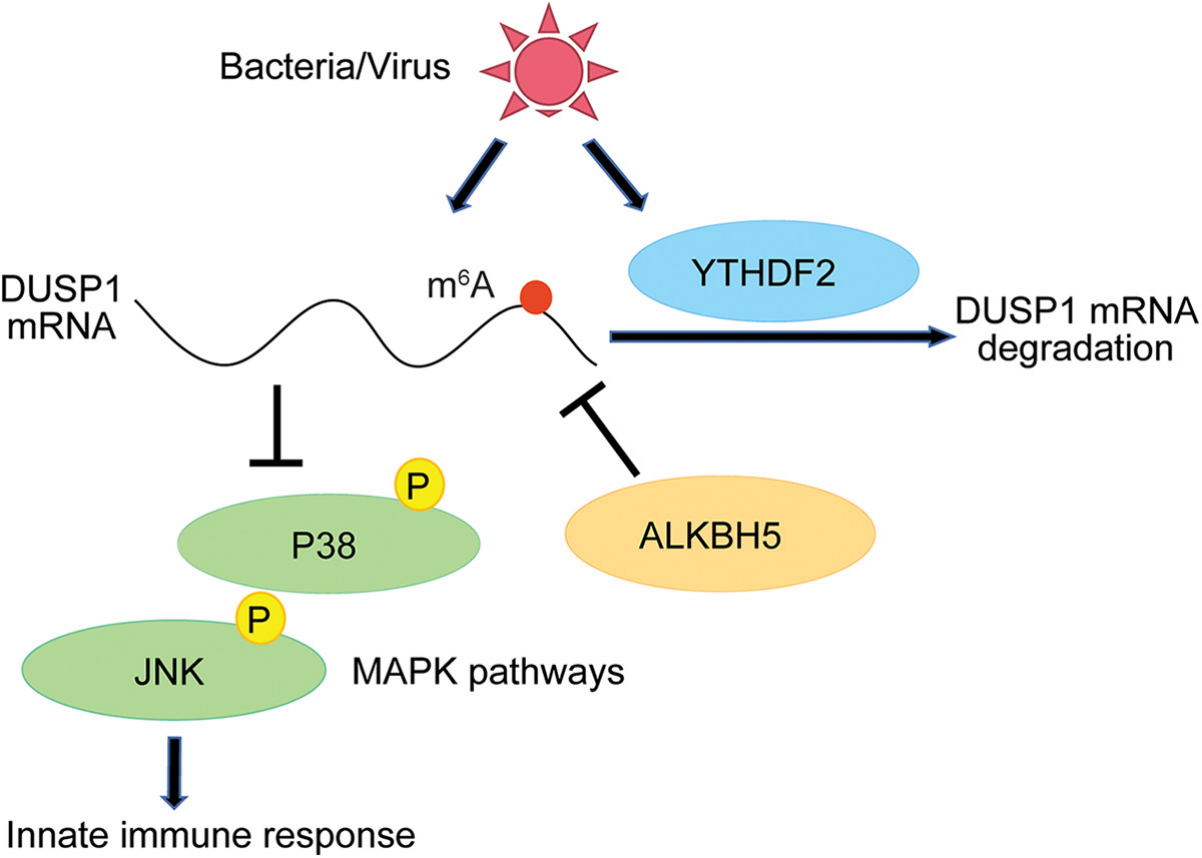
Working model of the regulation of DUSP1, MAPKs, and innate immune response genes by m^6^A and the m^6^A-related proteins YTHDF2 and ALKBH5 during pathogenic infections.

In conclusion, bacterial and viral infections activate MAPKs to induce innate immune response genes as well as a negative regulator of MAPKs, DUSP1, to avoid an excessive innate immune response. At the same time, numerous m^6^A writer proteins and the reader protein YTHDF2 are induced, leading to the hypermethylation of the DUSP1 transcript, which is targeted for YTHDF2-mediated degradation. This mechanism of the fine-tuned activation of MAPKs optimizes the induction of innate immune response genes during pathogenic infections (Fig. 6).

## DISCUSSION

The innate immune system is a complex cellular and molecular network in mammalian cells that serves as the first line of defense against pathogenic infections and is regulated by diverse cellular pathways (1). DUSP1 is a critical regulator of MAPK pathways, serving as a negative-feedback mechanism to prevent the excessive activation of these pathways (27, 28). In the context of pathogenic infections, the activation of MAPKs induces the expression of innate immune response genes as well as DUSP1, which prevents overreactive immune responses (29–31). Our results showed that the DUSP1 transcript was indeed induced during bacterial and viral infections, together with the activation of the ERK, JNK, and p38 MAPK pathways. At the same time, the m^6^A level of the DUSP1 transcript was significantly increased. During these processes, we observed only marginal increases of the m^6^A writer proteins METTL3 and METTL14 and no decrease of the m^6^A eraser proteins ALKBH5 and FTO, suggesting that the observed m^6^A increase in the DUSP1 transcript likely depended on preexisting writer proteins. Interestingly, despite the increased expression of the DUSP1 transcript during bacterial and viral infections, we failed to detect an increase of the DUSP1 protein. It is unclear whether the increased DUSP1 transcript m^6^A might affect its translation. In addition, it is unclear why the m^6^A level is increased in only some *de novo*-transcribed transcripts but not others. The specific mechanism involved in this selection process deserves further investigation. Nevertheless, the results of ALKBH5 knockdown experiments revealed that the m^6^A increase in the DUSP1 transcript targeted it for YTHDF2-mediated degradation. Importantly, YTHDF2 was significantly induced during bacterial infections, which maximized its negative regulation of DUSP1 transcript stability. Taken together, these results suggest that m^6^A and YTHDF2 are involved in fine-tuning the expression of the DUSP1 protein, an important regulator of innate immunity, during pathogenic infections.

The observed induction of the YTHDF2 protein is consistent with results from another study showing LPS induction of YTHDF2 expression (32). Interestingly, there was no obvious change in the YTHDF2 protein following HSV-1 infection, indicating the possible involvement of the bacterium-associated pattern recognition receptors in the induction of the YTHDF2 protein. However, it is possible that HSV-1 infection might have a YTHDF2 induction kinetic that is different from those of bacterial infections. Alternatively, HSV-1 might have evolved to prevent YTHDF2 induction as a mechanism to counter the innate immune response.

Our results showed that the m^6^A- and YTHDF2-mediated degradation of the DUSP1 transcript resulted in the enhanced activation of p38 and JNK. Both the p38 and JNK pathways activate transcriptional factors such as AP-1 and C/EBP that are essential for the expression of innate immune response genes. Indeed, the knockdown of DUSP1 or the m^6^A eraser ALKBH5 enhanced the expression of innate immune response genes, including IL-1β, CSF3, TGM2, and SRC, during bacterial or viral infections. We observed the robust induction of the IL-1β precursor by the HSV-1 ICP34.5 mutant but not the WT virus (see Fig. S2 in the supplemental material). It has been reported that the ICP34.5 protein can directly inhibit the TBK1 and eIF2α proteins to prevent the induction of innate immunity genes during HSV-1 infection (24, 25). Interestingly, activated MAPK pathways can promote HSV-1 replication by activating downstream transcriptional factors (33, 34). However, we showed that ALKBH5 knockdown inhibited HSV-1 replication, which was likely due to the m^6^A-mediated downregulation of DUSP1 and the subsequent activation of MAPK pathways resulting in the induction of the innate immune response. However, it is also possible that ALKBH5 and m^6^A might regulate HSV-1 replication through another mechanism in addition to targeting the DUSP1 transcript for degradation and activating MAPK pathways.

We have previously shown that a set of innate immune response genes are subjected to m^6^A modification and might be directly regulated by m^6^A, while another set of innate immune response genes might be indirectly regulated by m^6^A during bacterial and viral infections (13). In the current work, we have provided an example of the indirect regulation of innate immune response genes by m^6^A and YTHDF2 by mediating the stability of the DUSP1 transcript. In fact, DUSP1 is under the tight control of m^6^A and YTHDF2 during bacterial and viral infections. It can be speculated that other DUSP genes, which are involved in diverse cellular functions, could also be regulated by m^6^A and m^6^A-related proteins, which therefore deserves further investigation.

## MATERIALS AND METHODS

### Bacteria, viruses, and cells.

P. aeruginosa and C. diphtheriae were purchased from the ATCC. The herpes simplex virus 1 (HSV-1) F strain and the HSV-1 ICP34.5 mutant were obtained from Bernard Roizman (University of Chicago, Chicago, IL). The ICP34.5 mutant virus (R3616) was generated by deleting a 1-kb fragment containing both copies of the γ34.5 gene between the BstEII and StuI sites from the HSV-1 F strain genome (35). RAW264.7 cells were purchased from the ATCC and cultured according to the instructions of the vendor.

### Bacterial and viral infections.

RAW264.7 cells at 4 × 10^5^ cells per mL were infected with P. aeruginosa or C. diphtheriae at 10^7^ bacteria per mL or with WT HSV-1 or the HSV-1 ICP34.5 mutant at an MOI of 1. Cells were harvested at the indicated time points.

### m^6^A immunoprecipitation.

The isolation of m^6^A-containing fragments was performed as previously described (13, 36). Briefly, total RNA was extracted from cells using Tri reagent (catalog number T9424-200ML; Sigma-Aldrich) and fragmented using an RNA fragmentation kit (catalog number AM8740; Thermo Fisher). The successful fragmentation of RNA with sizes close to 100 nucleotides was validated using a bioanalyzer (2100 Bioanalyzer instrument; Agilent). Anti-m^6^A antibody (10 μg) (catalog number 202-003; Synaptic Systems) was incubated with a 30-μL slurry of Pierce protein A agarose beads (catalog number 20365; Thermo Fisher) by rotation in 250 μL of phosphate-buffered saline (PBS) at 4°C for 3 h. The beads were washed three times in cold PBS, followed by one wash in IP buffer containing 10 mM Tris-HCl at pH 7.4, 150 mM NaCl, and 1% Igepal CA-630 (catalog number I8896-50ML; Sigma-Aldrich). To isolate the m^6^A-containing fragments, 120 μg of fragmented total RNA was added to the antibody-bound beads in 250 μL of IP buffer supplemented with RNasin Plus RNase inhibitor (catalog number PRN2615; Promega), and the mixture was incubated at 4°C for 2 h. The beads were washed seven times with 1 mL IP buffer and eluted with 100 μL IP buffer supplemented with 6.67 mM m^6^A salt (catalog number M2780; Sigma-Aldrich) at 4°C for 1 h. A second elution was carried out, and the eluates were pooled before purification by 70% ethanol precipitation.

### siRNA knockdown.

siRNA silencing was performed by transfecting 2.5 pmol of each siRNA per well in a 12-well plate into RAW264.7 cells using Lipofectamine RNAi Max (catalog number 13778150; Thermo Fisher) according to the manufacturer’s instructions. Two days after transfection, the cells were monitored for the knockdown efficiency of the target gene by RT-qPCR and Western blotting. The following siRNAs were purchased from Sigma-Aldrich: siRNA 1 targeting DUSP1 (DUSP1 si1) (catalog number SASI_Mm02_00322441), DUSP1 si2 (catalog number SASI_Mm01_00056586), DUSP1 si3 (catalog number SASI_Mm01_00056587), ALKBH5 si1 (catalog number SASI_Mm01_00106232), ALKBH5 si2 (catalog number SASI_Mm02_00344968), ALKBH5 si3 (SASI_Mm01_00106233), and negative-control siRNA (NC) (siRNA universal negative control 1, catalog number SIC001-10NMOL).

### RNA stability assay.

Actinomycin D (10 μg/mL) (catalog number A9415-2MG; Sigma-Aldrich) was added to cells to inhibit transcription. RNA was isolated 0, 2, 4, and 6 h after actinomycin D treatment using TRIzol, and the transcripts were quantified by RT-qPCR.

### RT-qPCR for gene expression, RIP-qPCR for YTHDF2 RNA binding quantification, and MeRIP-qPCR for m6A sequencing (m^6^A-seq) validation.

Total RNA was isolated with Tri reagent (catalog number T9424-200ML; Sigma-Aldrich) according to the manufacturer’s instructions. Reverse transcription was performed with 1 μg of total RNA using a Maxima H Minus first-strand cDNA synthesis kit (catalog number K1652; Thermo Fisher). Quantitative PCR was done using SsoAdvanced universal SYBR green supermix (catalog number 1725271; Bio-Rad). Relative gene expression levels were obtained by normalizing the cycle threshold (*C_T_*) values to yield 2^−ΔΔ^*^CT^* values. For the validation of m^6^A-seq, eluted or input mRNA was subjected to RT-qPCR. Fold enrichment values were obtained by calculating the 2^−ΔΔ^*^CT^* value of the eluate relative to that of the input sample. The primers used for gene expression are as follows: 5′-CTGGTGGGTGTGTCAAGCAT-3′ (forward) and 5′-GAGGCAGTTTCTTCGCTTGC-3′ (reverse) for DUSP1, 5′-CCCTGAAGTACCCCATTGAA-3′ (forward) and 5′-GGGGTGTTGAAGGTCTCAAA-3′ (reverse) for β-actin, 5′-GAGTGTGGATCCCAAGCAAT-3′ (forward) and 5′-ACGGATTCCATGGTGAAGTC-3′ (reverse) for IL-1β, 5′-CCGGTACCCTCTCCTGTTGTGTTTA-3′ (forward) and 5′-AACTCGAGCTAAAAAGGAGGACGGC-3′ (reverse) for CSF3, 5′-AAGAGCTCCAAACAAGGTCTGCCTT-3′ (forward) and 5′-AACTCGAGACGTGCCATATAAGCAC-3′ (reverse) for TGM2, 5′-AAGGTACCCTGCCAGGCCAGACCAA-3′ (forward) and 5′-AACTCGAGCCAGCCTTGACCCTGAG-3′ (reverse) for SRC, 5′-ACGGTTTACTACGCCGTGTT-3′ (forward) and 5′-TGTAGGGTTGTTTCCGGACG-3′ (reverse) for US6, 5′-GACGAACATGAAGGGCTGGA-3′ (forward) and 5′-CGACCTGTTTGACTGCCTCT-3′ (reverse) for VP16, 5′-CCCACTATCAGGTACACCAGCTT-3′ (forward) and 5′-CTGCGCTGCGACACCTT-3′ (reverse) for ICP0, and 5′-GCATCCTTCGTGTTTGTCATTCTG-3′ (forward) and 5′-GCATCTTCTCTCCGACCCCG-3′ (reverse) for ICP27.

### Western blotting.

Protein samples were lysed in Laemmli buffer, separated by SDS-PAGE, and transferred to a nitrocellulose membrane (37). The membrane was blocked with 5% milk and then incubated with primary antibody to glyceraldehyde-3-phosphate dehydrogenase (GAPDH) (catalog number 5174S; Cell Signaling Technology [CST]), p38 (catalog number 8690S; CST), p-p38 (catalog number 4511S; CST), ERK (catalog number 4695S; CST), p-ERK (catalog number 4370S; CST), JNK (catalog number 9252S; CST), p-JNK (catalog number 4668S; CST), DUSP1 (catalog number NBP2-67909; Novus), IL-1β (catalog number AB-401-NA; R&D Systems), or ALKBH5 (catalog number HPA007196; Sigma) overnight at 4°C. The membrane was washed with Tris-buffered saline (TBS)–Tween (TBS-T) and probed with a secondary antibody conjugated to horseradish peroxidase (HRP). After further washing with TBS-T, the blot was visualized using SuperSignal West Femto maximum-sensitivity substrate (catalog number 34096; Thermo) and imaged on a ChemiDoc MP imaging system (catalog number 12003154; Bio-Rad).

### Nuclear run-on assay.

Nuclear run-on assays were conducted as previously described (38).

### RNA immunoprecipitation assay.

RNA immunoprecipitation (RIP) assays were conducted as previously described (39).
