# Supplementary figures and images for: N6-Methyladenosine and Reader Protein YTHDF2 Enhance the Innate Immune Response by Mediating DUSP1 mRNA Degradation and Activating Mitogen-Activated Protein Kinases during Bacterial and Viral Infections
Source: mBio. 2023 Jan 10;14(1):e03349-22. doi: 10.1128/mbio.03349-22 (PMC9973302; doi:10.1128/mbio.03349-22)

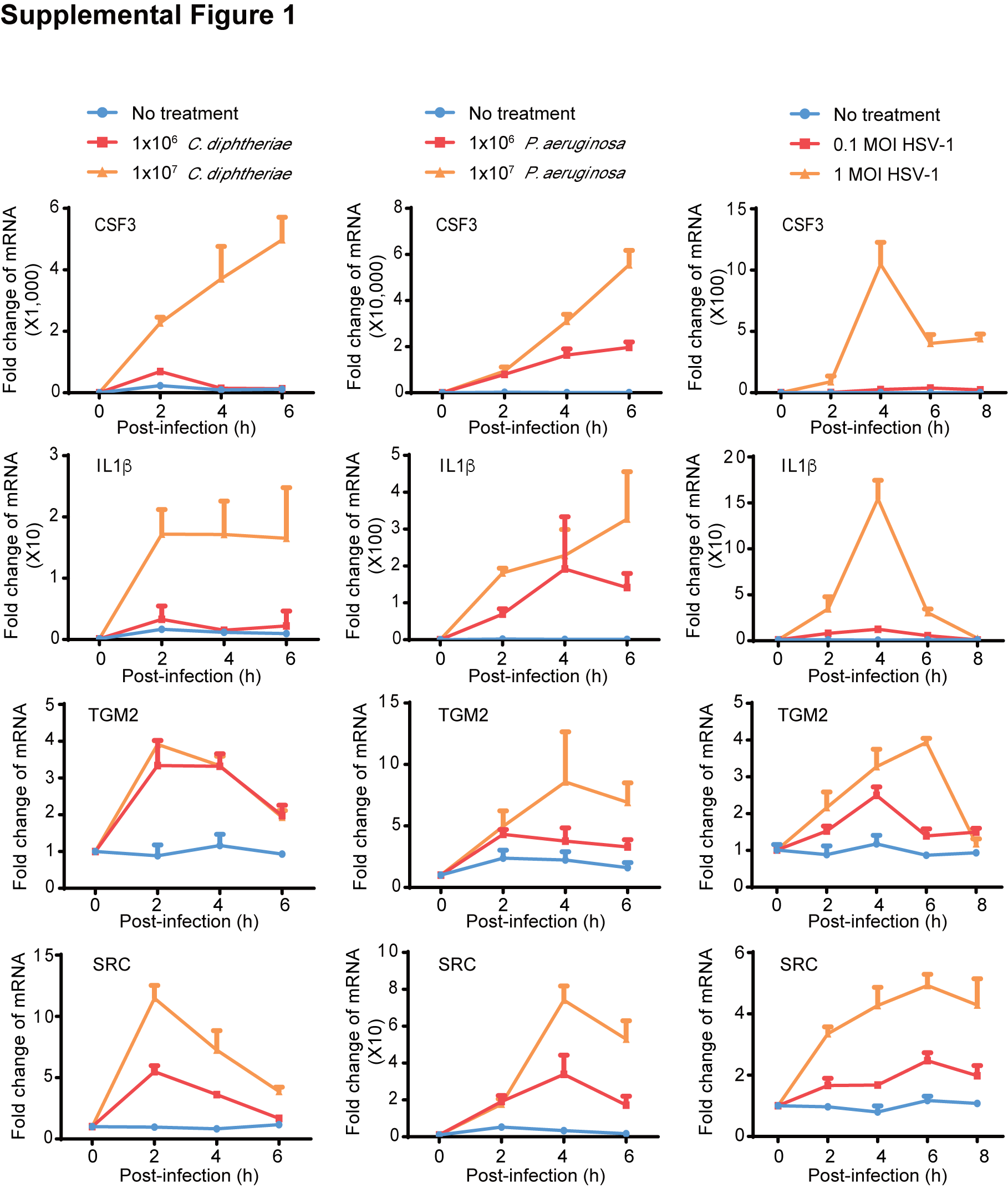

Supplement: FIG S1 [file mbio.03349-22-s0001.tif]

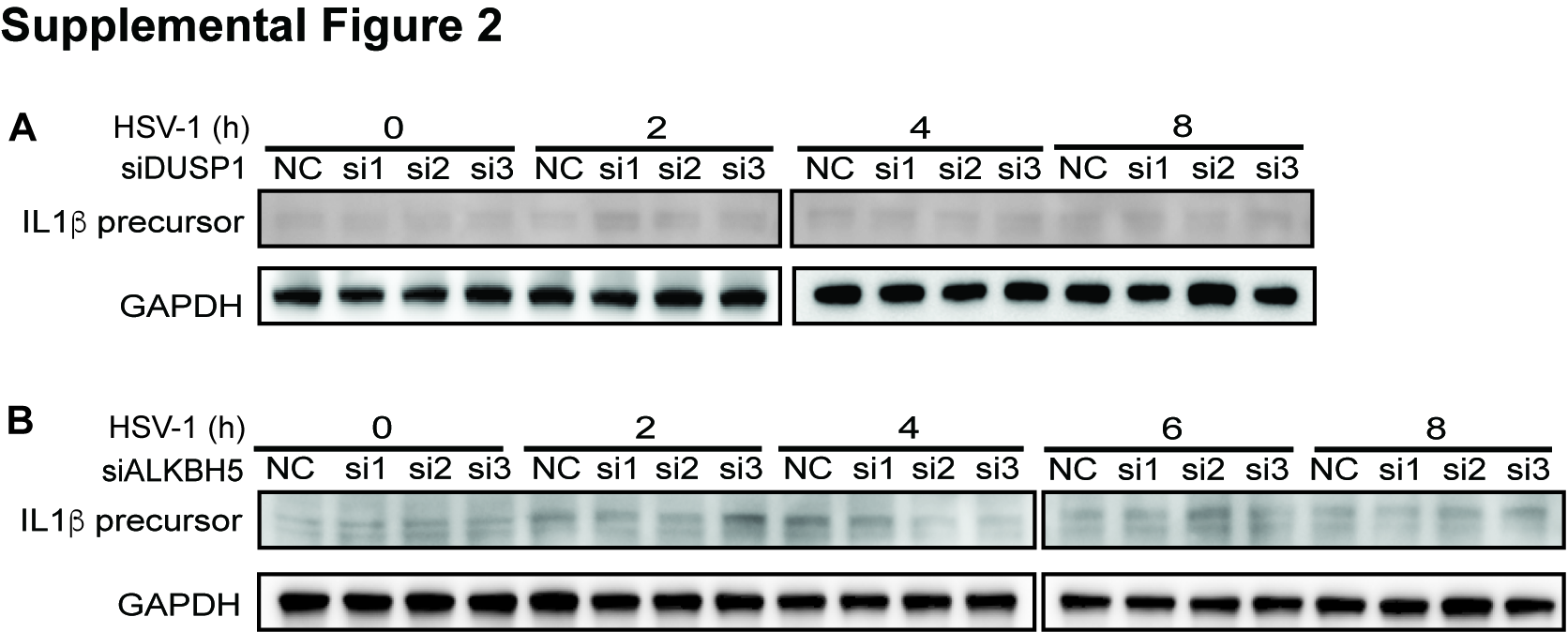

Supplement: FIG S2 [file mbio.03349-22-s0002.tif]

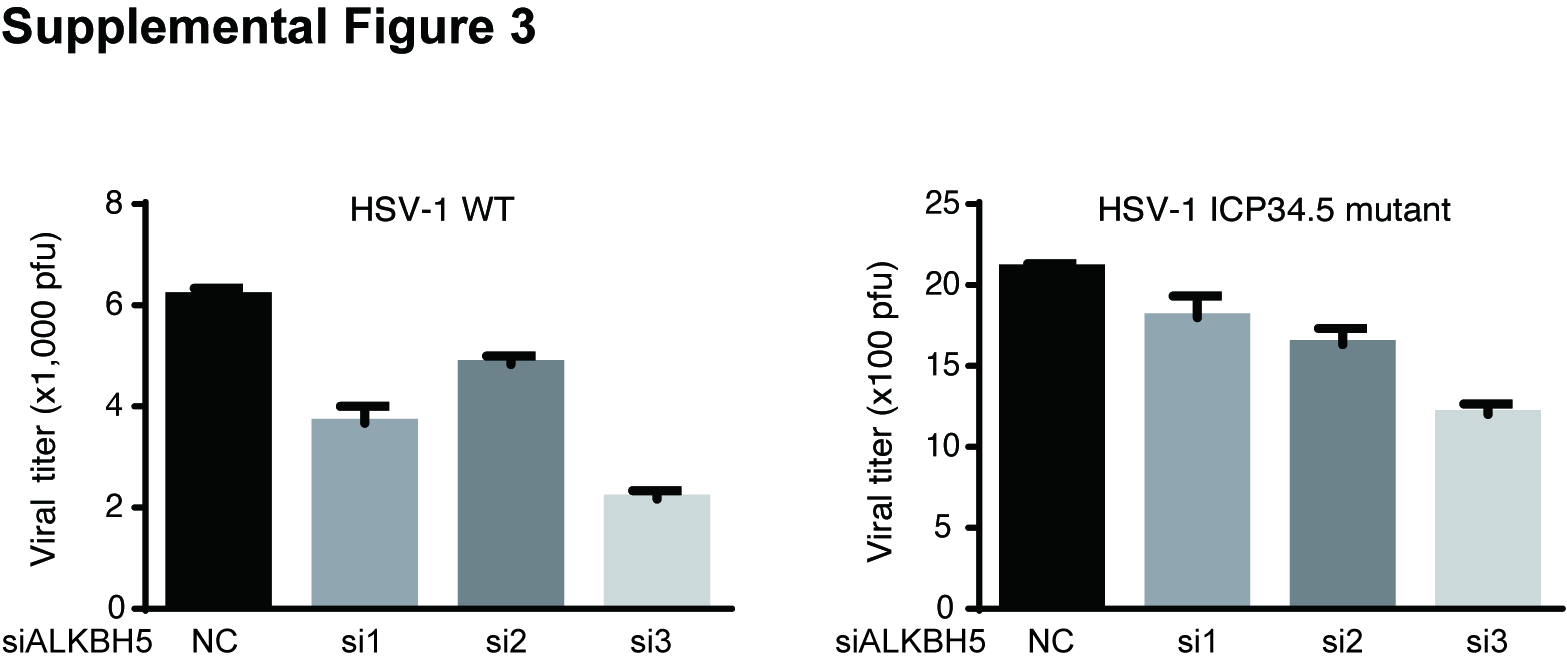

Supplement: FIG S3 [file mbio.03349-22-s0003.tif]
